# Hermansky-Pudlak Syndrome: Identification of *Novel* Variants in the Genes *HPS3*, *HPS5,* and *DTNBP1* (HPS-7)

**DOI:** 10.3389/fphar.2021.786937

**Published:** 2022-01-19

**Authors:** Doris Boeckelmann, Mira Wolter, Katharina Neubauer, Felix Sobotta, Antonia Lenz, Hannah Glonnegger, Barbara Käsmann-Kellner, Jasmin Mann, Stephan Ehl, Barbara Zieger

**Affiliations:** ^1^ Department of Pediatrics and Adolescent Medicine, Division of Pediatric Hematology and Oncology, Faculty of Medicine, Medical Center—University of Freiburg, Freiburg, Germany; ^2^ Department of Ophthalmology, Saarland University Medical Center, Homburg, Germany; ^3^ Institute for Immunodeficiency, Center for Chronic Immunodeficiency (CCI), Faculty of Medicine, Medical Center—University of Freiburg, Freiburg, Germany

**Keywords:** Hermansky-Pudlak syndrome (HPS), HPS-3, HPS-5, HPS-7, BLOC-1, BLOC-2

## Abstract

Hermansky-Pudlak syndrome (HPS), a rare heterogeneous autosomal recessive disorder, is characterized by oculocutaneous albinism (OCA) and a bleeding diathesis due to a defect regarding melanosomes and platelet delta (δ)-granule secretion. Interestingly, patients with HPS type 2 (HPS-2) or HPS type 10 (HPS-10) present additionally with an immunological defect. We investigated three patients (IP1, IP2, and IP3) who suffer from a bleeding diathesis. Platelet aggregometry showed impaired platelet function and flow cytometry revealed a severely reduced platelet CD63 expression hinting to either a defect of platelet delta granule secretion or a decreased number of delta granules in these patients. However, only IP3 presents with an apparent OCA. We performed panel sequencing and identified a homozygous deletion of exon 6 in *DTNBP1* for IP3*.* Western analysis confirmed the absence of the encoded protein dysbindin confirming the diagnosis of HPS-7. Interestingly, this patient reported additionally recurrent bacterial infections. Analysis of lymphocyte cytotoxicity showed a slightly reduced NK-degranulation previously documented in a more severe form in patients with HPS-2 or HPS-10. IP1 is carrier of two compound heterozygous variants in the *HPS3* gene (c.65C > G and c.1193G > A). A homozygous variant in *HPS5* (c.760G > T) was identified in IP2. The novel missense variants were classified as VUS (variant of uncertain significance) according to ACMG guidelines. For IP1 with the compound heterozygous variants in *HPS3* a specialized ophthalmological examination showed ocular albinism. *HPS3* and *HPS5* encode subunits of the BLOC-2 complex and patients with HPS-3 or HPS-5 are known to present with variable/mild hypopigmentation.

## Introduction

Hermansky-Pudlak syndrome (HPS) which was first described by Hermansky and Pudlak (Hermansky and Pudlak 1959) has a prevalence of 1–9 per 1,000,000 individuals (Christensen et al., 2017). The key characteristics of HPS include oculocutaneous albinism (OCA) and a bleeding tendency. Typically, the patients present with congenital nystagmus, iris transillumination, decreased visual acuity, and reduced skin/hair pigmentation (Gahl et al., 1998). Bleeding symptoms may manifest as epistaxis, petechiae, extensive bruising or even serious post-traumatic or perioperative complications. To date, 11 types of HPS have been described (HPS-1- HPS-11) explaining some of the clinical variability that relates to the biological functions of the impaired protein complex (Huizing et al., 2020; Pennamen et al., 2020). Malfunctioning of lysosome-related organelles such as melanosomes and platelet δ-granules causes HPS, as they are essential for granule transport (Vincent et al., 2009). Platelet δ-granules secrete serotonin, calcium, ADP, and polyphosphate, therefore, enhancing platelet adhesion and activation (Bowman et al., 2019). Currently, 11 genes associated with HPS (*HPS1, AP3B1, HPS3, HPS4, HPS5, HPS6, DTNBP1, BLOC1S3, BLOC1S6, AP3D1, and BLOC1S5*) have been reported. These genes encode either for the multi-protein complexes BLOC, (biogenesis of lysosome-related organelles complex) or AP-3 (adaptor protein-3).

BLOC-1 comprises the gene products of *DTNBP1* (HPS-7)*, BLOC1S3* (HPS-8)*, BLOC1S6* (HPS-9), and *BLOC1S5* (HPS-11) (Huizing et al., 2020; Pennamen et al., 2020). *DTNBP1* (HPS-7) is located on chromosome 6 (6p22.3) and comprises ten exons. The gene codes for 351 amino acid polypeptides (MW 39.5 kD). To our knowledge, only seven patients with four different pathogenic genetic variants in *DTNBP1* (HPS-7) have been described (Li et al., 2003; Lowe et al., 2013; Bryan et al., 2017; Lasseaux et al., 2018; Bastida et al., 2019). These patients exhibit a characteristic phenotype of bleeding diathesis and hypopigmentation (OCA). For these patients signs of immunodeficiency or pulmonary fibrosis were not reported, however, the number of patients described with HPS-7 is very low.

BLOC-2 subunits are encoded by *HPS3*, *HPS5,* and *HPS6* (Di Pietro et al., 2004; Gautam et al., 2004). *HPS3* is located on chromosome 3 (3q24) and *HPS5* on chromosome 22 (22q.12.2), respectively. *HPS3* (MW 11.7kD) encompasses 17 exons coding for a 1,004 amino acid polypeptide. In 2001 the first patients with HPS-3 were described (Anikster et al., 2001; Huizing et al., 2001). *HPS5* (127.4 kD) codes for a 1,129 amino acid polypeptide and comprises 23 exons. Zhang et al. identified the first pathogenic variant in human *HPS5*, which is orthologue to ru2, the gene mutated in a HPS mimicking mouse model (Zhang et al., 2003). Individuals with pathogenic variants in BLOC-2 seem to present a milder HPS phenotype causing a moderate bleeding diathesis and an OCA with variable hypopigmentation (Nurden et al., 2020).

BLOC-3 encompasses the gene products of *HPS1* and *HPS4* (Wei 2006). Deficiencies in these proteins are associated with a more severe bleeding diathesis, OCA, and serious complications such as pulmonary fibrosis and granulomatous colitis (Huizing et al., 2020; Nurden et al., 2020).

HPS-2 and HPS-10 are caused by variants in *AP3B1* and *AP3D1*, respectively, which constitute the adaptor protein-3 (AP-3) complex. Affected patients present with a bleeding diathesis, OCA, and immunodeficiency due to impaired cytotoxic activity of T-lymphocytes and/or natural killer (NK) cells (Fontana et al., 2006; Ammann et al., 2016; Mohammed et al., 2018). HPS-2 patients are at risk to develop pulmonary fibrosis in childhood (Hengst et al., 2018). One patient with HPS-2 developed hemophagocytic lymphohistiocytosis (HLH) (Enders et al., 2006). For patients with HPS-2, the risk to develop HLH is lower than for patients with Griscelli or Chediak-Higashi syndrome (Jessen et al., 2013).

Here, we report *novel* genetic alterations in *HPS3, HPS5,* and *DTNBP1* (HPS-7) in patients with a platelet delta granule secretion defect and differently pronounced OCA. Interestingly, we also document a mild NK cell degranulation defect in HPS-7, potentially implicating BLOC-1 also in immune functions.

## Materials and Methods

### Patients

#### Index Patient 1

The six-year-old girl (ethnic origin: European) presented with frequent epistaxis (once a month) and bruising. Previous surgery had not been performed. Cutaneous albinism was not apparent. After the molecular genetic analysis had identified compound heterozygous variants in the gene *HPS3*, a specialized ophthalmological examination was initiated and showed ocular albinism (atypical albino-VEP) with normal visual acuity (no nystagmus). The girl’s mother exhibits prolonged menstrual bleeding. Her father and older sister did not show any bleeding symptoms. The parents are not consanguine.

#### Index Patient 2

The 45-year-old woman (ethnic origin: Arabic) had a history of extensive bruising, epistaxis, menorrhagia, postoperative bleeding (teeth extraction, liposuction), bleeding after deliveries, joint hemorrhages, microhematuria, and impaired wound healing/increased scarring after surgery. She has eight children and suffered from increased bleeding during childbirth. Therefore, she had received red blood cell and platelet concentrates each time. The symptoms of menorrhagia improved significantly after therapy with tranexamic acid and desmopressin. Cutaneous albinism was not apparent. She and her husband are consanguine, however, her husband does not present any bleeding symptoms. Some of their 8 children seem to exhibit only very mild bleeding symptoms, none of them is clinically as severely affected as their mother. We performed panel sequencing for IP2 and two of her children (daughter and son).

#### Index Patient 3

The 60-year-old woman (ethnic origin: European, Portuguese descent) presented with OCA and frequent gingival bleeding. Furthermore, she had experienced prolonged bleeding after skin excision and adenoma resection. She did not show signs of colitis. She suffers from asthma. She reported that she has always suffered from severe respiratory infections and recurrent skin furuncles. The patient does not have children. Her consanguine parents are deceased. Her brother had OCA and recurring epistaxis. At the age of 54 ears, he died due to liver cirrhosis (caused by a hepatitis B infection) and due to internal bleeding.

### Platelet Count and Platelet Aggregometry Analysis

Platelet count was measured using an automated cell counter (Sysmex KX-21 N, Norderstedt, Germany). Platelet-rich plasma (PRP) and platelet-poor plasma (PPP) were obtained by centrifugation of citrate-anticoagulated blood samples. Using the APACT 4004 (LABiTec, Ahrensburg, Germany), platelet aggregometry analyses were performed after stimulation with collagen (2 and 10 μg/ml; Takeda, Linz, Austria), adenosine diphosphate (ADP; 4 and 10 μmol/L; Sigma-Aldrich, St. Luis, MO, United States), epinephrine (8 and 16 μmol/L; Sanofi-Aventis, Frankfurt, Germany) and ristocetin (1.2 mg/ml; American Biochemical and Pharmaceutical LTD., Frankfurt, Germany).

### Flow Cytometry Analyses

Flow cytometry analyses were performed using FACSCalibur (Becton Dickinson, Heidelberg, Germany) (Lahav et al., 2002). Diluted PRP aliquots (5 × 10^7^ platelets/ml) were fixed and stained with FITC-labeled monoclonal surface antibody against CD41 (GPIIb/IIIa-complex), CD42a (GPIb/IX) and CD42b (GPIb) (Coulter, Immunotech, Marseille, France). FITC-labeled anti-VWF (Bio-Rad AbD Serorech, Puchheim, Germany) and Alexa Fluor 488-labeled anti-fibrinogen (Invitrogen, Waltham, MA United States) was used to stain the platelets. In the presence of 1.25 mM Gly-Pro-Arg-Pro (Bachem, Bubendorf, Switzerland) diluted PRP (5 × 10^7^ platelets/ml) was stimulated with a number of concentrations of thrombin (0, 0.05, 0.1, 0.2, 0.5, and 1 U/ml; Siemens Healthineers, Marburg, Germany) to conduct the CD62 and CD63 expression analyses. Additionally, the platelets were stained with monoclonal FITC-labeled anti-CD62 (P-selectin) and anti-CD63 antibodies (lysosomal membrane-associated glycoprotein 3, LAMP-3; Immunotech, Marseille, France). Data of patients and controls (day control and 20 independent measurements from 10 controls as mean ± standard error of the mean, SEM) were analyzed using GraphPad Prism software (version 8, San Diego, CA, United States).

### Molecular Genetic Analyses

Informed consent for molecular genetic analysis was obtained for each patient and the investigated family members. To extract genomic DNA from EDTA blood, we used standard procedures and the Blood and Cell Kit by Qiagen (Qiagen GmbH, Hilden, Germany). For index patients panel sequencing (95 genes including all 11 HPS genes; Supplementary Material S1 gene list) was performed using a custom-designed Nextera Rapid Enrichment Kit (Illumina) followed by sequencing on a MiSeq (Illumina). The average sequencing depth overall genes for the 3 patients investigated was 98% for 20x and 91% for 100x, respectively. SeqPilot (JSI medical systems) was used for data analyses. The variants were exported and filtered by allele frequency and serious consequences. We utilized supporting software ALAMUT^®^(v.2.15), pathogenicity prediction (SIFT, MutTaster, PolyPhen2, and CADD), occurrence in population and disease databases (HGMD public version, Huizing HPS Mutation update (Huizing et al., 2020)) in order to classify the variants. These analyses were performed in accordance with the ACMG (American College of Medical Genetics) guidelines (Richards et al., 2015). For segregation analysis Sanger sequencing was conducted.

### cDNA Sequencing Using Reverse Transcripted Platelet-Derived mRNA (for IP3)

Total RNA was isolated from washed human platelets using 2 ml TRIzol^®^ reagent (Thermo Fisher Scientific). Single strand cDNA synthesis was generated with SuperScript^®^ III Reverse Transcriptase (Thermo Fisher Scientific). Amplification was performed using specific primers (fw: TGC​AGC​AGG​ATT​TCA​CCT​CC; rev: ATC​TGC​TCC​AGC​ATG​TCC​AC) covering the coding region from exon 1 to exon 9 of *DTNBP1.*

### Platelet Preparation and Immunoblotting (for IP3)

Platelet-rich plasma (PRP) of the patients and a healthy volunteer was obtained by centrifugation of citrate-anticoagulated whole blood (120 × g for 10 min). Platelets were then isolated from PRP by gel-filtration using a Sepharose CL-2B (GE Healthcare Life Sciences) column, eluted with Tyrode buffer (140 mM NaCl, 2.7 mM KCl, 0.42 mM NaH_2_PO_4_, 12 mM NaHCO_3_, 5.5 mM glucose, and 5 mM HEPES; pH 7.4). Platelets were centrifuged at 1,200 × g for 10 min and lysed in lysis buffer (5 mM Tris-HCl, pH 7,4, 50 mM NaCl, 0,25 mM MgCl_2_, 0.1% Nonidet P40, 10% glycerol including protease inhibitor cocktail; Roche cOmplete, Merck). Protein content was determined with Pierce BCA Protein Assay Kit (Thermo Fisher Scientific) and adjusted to 10 µg. Denatured platelet lysates were separated by sodium dodecyl sulfate-polyacrylamide gel electrophoresis (SDS-PAGE, Invitrogen) and blotted onto Hybond-P polyvinylidene difluoride membrane (PVDF, Amersham, GE Healthcare Life Sciences). Membranes were blocked with 5% milk powder in TBST (20 mM Tris, 140 mM NaCl, 0.1% Tween; pH 7.6) probed with anti-dysbindin antibody (dilution 1:2.000; Abcam) and detected using horseradish peroxidase (HRP)-conjugated goat anti-rabbit (dilution 1:10.000; Cell Signaling Technology) and enhanced chemiluminescence solution (Amersham detection reagent, GE Healthcare Life Sciences). For loading control, the same blot was incubated with anti-GAPDH (glyceraldehyde-3-phosphate dehydrogenase; dilution 1:300.000; Abcam) and HRP-coupled goat anti-mouse (dilution 1:10.000; Dianova).

### Analysis of NK Cell and CTL Degranulation (for IP3)

NK cell degranulation was analyzed as described (Bryceson et al., 2012). Briefly, fresh NK cell degranulation was assessed by stimulation of isolated peripheral blood mononuclear cells (PBMCs) with K562 target cells followed by flow cytometric analysis of CD107a surface expression. For evaluation of activated NK cell and CTL degranulation, PBMCs were cultured in the presence of Phytohemagglutinin (PHA) and Interleukin-2 (IL-2) for 48 h at 37°C prior to stimulation with K562 target cells and anti-CD3/CD28 beads.

## Results

### Platelet Count, In-Vivo Bleeding Time, and Platelet Function Analysis

All three index patients presented with normal platelet counts (234 × 10^9^/L, 173 × 10^9^/L, and 189 × 10^9^/L, respectively). The *in-vivo* bleeding time (Ivy) was severely prolonged (IP1 and IP3 > 15 min, IP2 > 8 min (norm < 6 min)). Platelet aggregation was severely impaired after stimulation with collagen (2 μg/ml) and epinephrine (8 μmol/L) for all index patients. Impaired aggregation after stimulation with low dose ADP (4 μmol/L) was seen in IP1 and IP2. Agglutination after stimulation with ristocetin (1.2 mg/ml) was normal (Table 1).

**TABLE 1:** Platelet aggregometry analyses (LTA). Data are presented as maximal aggregation/agglutination in % compared to normal control levels.

Stimulation	IP1 [%]	IP2 [%]	IP3 [%]	Norm [%]
Collagen (2 μg/ml)	**11**	**26**	**29**	>70
Collagen (10 μg/ml)	75	76	76	>70
Ristocetin (1,2 mg/ml)	84	94	89	>85
ADP (4 μmol/L)	**57**	**68**	77	>70
ADP (10 μmol/L)	86	-	77	>70
Epinephrine (8 μmol/L)	**50**	**15**	**46/37**	>70
Epinephrine (16 μmol/L)	71	**22**	**44**	>70

Reduced values are given in bold.

**FIGURE 1:**
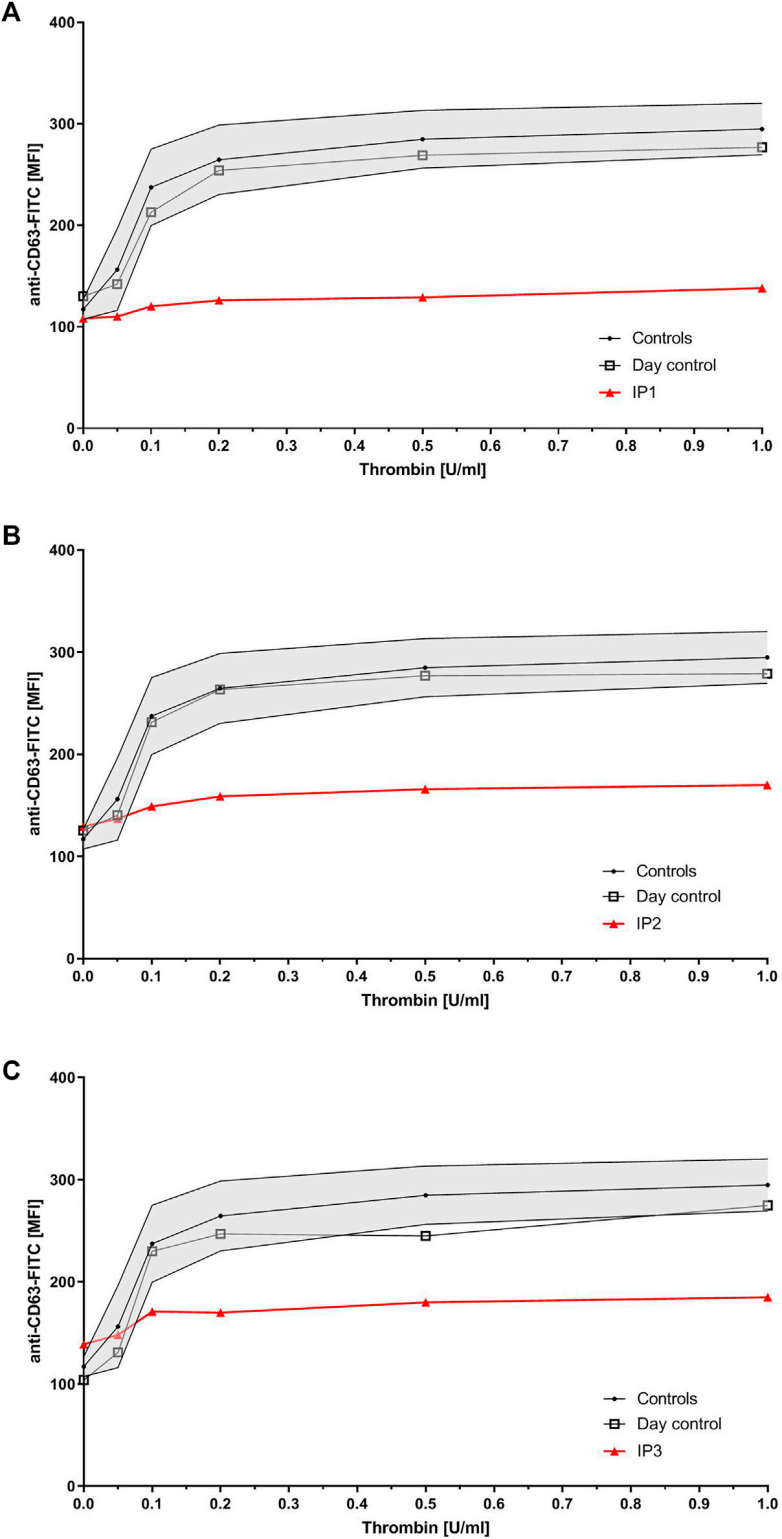
Platelet granule secretion stimulated with thrombin (concentrations: 0, 0.05, 0.1, 0.2, 0.5, and 1.0 U/ml) for all three index patients using flow cytometry. Severely impaired δ-granule secretion indicated by reduced platelet CD63 expression in IP1 **(A)**, IP2 **(B)**, and IP3 **(C)** compared to the healthy controls/day control. Data are expressed as logarithmic arbitrary units (logAU) of anti-CD63-stained unstimulated and thrombin-stimulated platelets.

Flow cytometry analysis revealed severely reduced CD63 expression for all index patients (Figure 1). All of them showed normal values for expression of CD62, CD42a, CD42b, CD41, fibrinogen binding, and VWF-binding (data not shown). IP1’s sister presented with only slightly reduced CD63 expression, while her mother`s expression was borderline low. Flow cytometry was not performed for the father. Six of IP2`s children presented with borderline low and two with a slightly reduced CD63 expression.

### Molecular Genetic Analysis, cDNA Sequencing, and Immunoblotting

In IP1 two *novel* heterozygous missense variants in *HPS3* (NM_032383.3) were identified (c.65C > G; p.Pro22Arg and c.1193G > A; p.Cys398Tyr). Family genotyping confirmed compound heterozygosity. Father and sister of IP1 are heterozygous carriers of the c.65C > G variant, whereas the mother is a carrier of the c.1193G > A variant. Since the index patient lacked apparent cutaneous albinism we confirmed both variants to be germline by genotyping buccal swab DNA. The c.65C > G variant is absent from GnomAD v2.1 (https://gnomad.broadinstitute.org/) and EVS v.0.0.30 (https://evs.gs.washington.edu/EVS/) databases (ACMG Classification: uncertain significance [PM1, PM2]). The variation shows a moderately conserved nucleotide (phyloP: 4.79 [−19.0, 10.9]. *In silico* pathogenicity prediction (PP) is predominantly disease causing (SIFT, tolerated; MutationTaster, disease causing; PolyPhen2, probably damaging, CADD score 29.1). The second variant c.1193G > A is reported once in the GnomAD database in a heterozygous state and absent from EVS (ACMG Classification: uncertain significance [PM1, PM2, PP3]). *In silico* PP is concordant disease causing and CADD score is 29.1.

In IP2 a *novel* homozygous missense variant (c.760G > T; p.Val254Phe) in *HPS5* (NM_181507.1) was identified. The patient’s husband showed wild type sequence at this position. NGS and Sanger sequencing revealed that all of her eight children were heterozygous carriers for this *HPS5* variant as expected. The variant c.760G > T is reported six times in the GnomAD database in a heterozygous state (all counts in South Asian population) and absent from EVS (ACMG Classification: uncertain significance [PM1, PM2, PP3]). *In silico* PP is concordant disease causing and CADD score is 28.1. Additionally we identified a variant (NM_018668.5:c.1780A > G) in the gene *VPS33B* in a heterozygous state*.* This variant was also detected in the NGS analysis of her daughter. According to OMIM alterations in the gene *VPS33P* are autosomal recessive associated with ARC (Arthrogryposis, renal dysfunction, and cholestasis) and α-granule deficiency.

**FIGURE 2:**
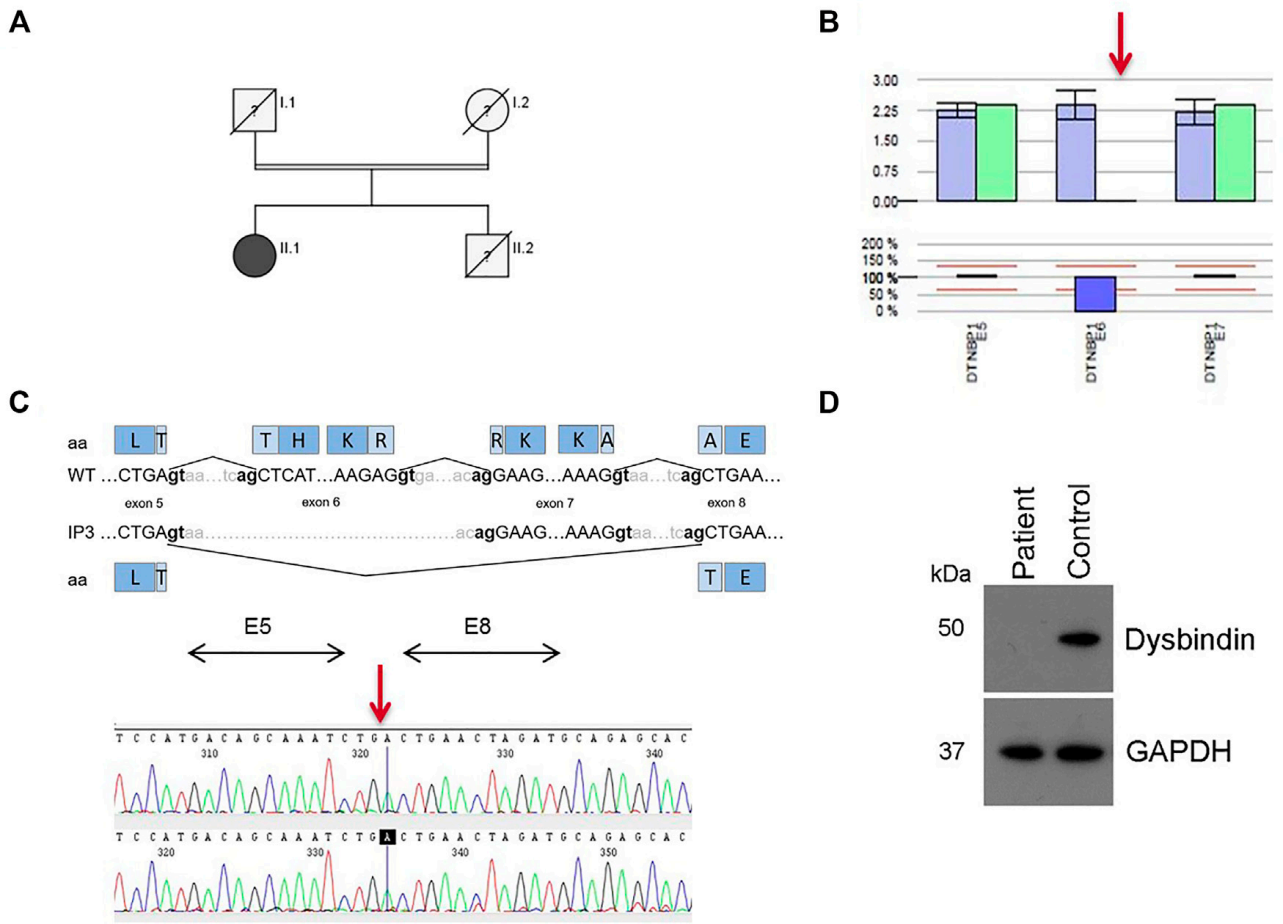
Results for patient IP3 (HPS-7). **(A)** Pedigree for IP3 (II.1). Her consanguine parents and her brother are already deceased, her brother presented with OCA and bleeding disorder. **(B)** Bioinformatics software SeqPilot CNV analysis indicates a homozygous deletion of exon 6 in *DTNBP1*. **(C)** Upper Panel: Schematic representation of the normally spliced mRNA (WT) and the aberrantly spliced mRNA of IP3. Lower Panel: cDNA sequencing of platelet derived *DTNBP1* mRNA shows deletion of exon 6 and 7 in IP3. **(D)** Dysbindin expression in gel-filtrated platelets of the patient (IP3) and a healthy volunteer performed by Western analysis: Dysbindin is not expressed in patient’s platelets. GAPDH (37 kDa) was used as loading control (lower bands).

IP3 showed a *novel* homozygous deletion of exon 6 in *DTNBP1* (Figure 2B). Exon 6 could not be amplified from genomic DNA using PCR analysis. We performed cDNA sequencing out of platelet derived mRNA and confirmed the absence of exon 6. Interestingly, the small exon 7 was spliced out. It seems that the spliceosome used the acceptor splice site of exon 8. This would result in an in-frame loss of 52 amino acids in the middle of the expected protein (p.H120_A171del). (Figure 2C). To investigate if the aberrant mRNA is leading to detectable dysbindin we performed Western blot analysis. Dysbindin, which is encoded by *DTNBP1,* was not detectable in platelets (Figure 2D). The antibody used recognizes the C-terminal sequence within human dysbindin (aa 251-301). However, it is possible that the protein will fold incorrectly and in addition protein degradation will occur. Genetic alterations of all patients are summarized in Table 2.

**TABLE 2:** OCA phenotype and genetic variants identified using NGS for each patient.

ID	OCA	Gene	Variant	Protein	Occurrence in database/dbSNP ID/MAF gnomAD (v2.1)	PP	ACMG classification
IP1	OA	*HPS3*	c.[65C > G]; [1193G > A]	p.Pro22Arg	-	CADD: 29.1	VUS (PM1, PM2)
						MutTaster, PolyPhen2	
				p.Cys398Tyr	rs1360046176	CADD: 29.3	VUS (PM1, PM2, PP3)
					MAF: 0,0007%	SIFT, MutTaster, PolyPhen2	
IP2	n.a.	*HPS5*	c.[760G > T]; [760G > T]	p.Val254Phe	rs752603589	CADD: 28.1	VUS (PM1, PM2, PP3)
					MAF:0.004%	SIFT, MutTaster, PolyPhen2	
IP3	OCA	*DTNBP1*	c.(355 + 1_356–1)_(488 + 1_489–1)del; c.(355 + 1_356–1)_(488 + 1_489–1)del	no dysbindin expression^1^	-		P (PVS1, PM1, PM2)

Transcripts: *HPS3* (NM_032383.3), *HPS5* (NM_181507.1), *DTNBP1* (NM_032122.4). Abbr.: OCA, oculocutaneous albinism; OA, ocular albinism; n.a., not apparent; PP, *in silico* pathogenicity prediction; VUS, variant of uncertain significance; P, pathogenic.

1 Western Blot analysis.

### Analysis of Lymphocyte Cytotoxicity

**FIGURE 3:**
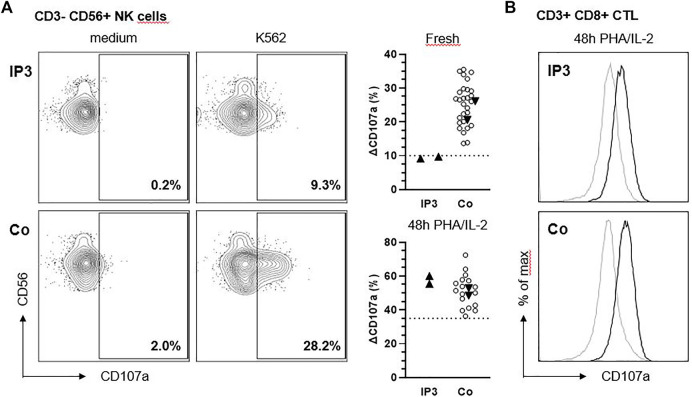
NK cell degranulation of IP3 is slightly reduced in response to K562. **(A)** *Ex vivo* degranulation of CD3^−^ CD56^+^ NK cells from IP3 and a healthy day control (Co.) after incubation with medium (left panel) or NK-sensitive K562 target cells (middle panel) assessed by flow cytometric analysis of CD107a surface expression. Graphs in the right panel show ∆CD107a for the patient and healthy day controls (solid triangles) pooled from two independent experiments assessing fresh and activated (PHA/IL-2 pre-culture for 48 h) NK cell degranulation. ∆CD107a was calculated as the percentage of NK cells expressing CD107a after stimulation with K562 minus the percentage of NK cells expressing CD107a after incubation with medium. Blank circles represent historical controls (*n* = 28,17). The dashed line indicates the diagnostic cutoff below which NK cell degranulation is considered abnormal and equals the 10th percentile of a reference range. **(B)** Degranulation of CD3^+^ CD8^+^ CTL after incubation with medium (grey line) or anti-CD3/CD28 beads (black line).

We analyzed the degranulation capacity of NK cells and cytotoxic T lymphocytes (CTL) from IP3 using CD107 degranulation assays. “Fresh” NK cell degranulation in response to K562 target cell stimulation was slightly impaired compared to more than 30 historical controls and additional day controls in two independent experiments (Figure 3A). Following IL-2 stimulation for 48h, the degranulation of patient NK cells increased to normal values. Moreover, degranulation of PHA/IL-2 blasts following stimulation with anti-CD3/28 was in the normal range (Figure 3B).

## Discussion

We report three patients with a severely impaired δ-granule secretion. NGS identified genetic alterations in three genes associated with different types of HPS. For IP3 the diagnosis of the rare HPS-7 was made after extensive investigations (cDNA sequencing, Western blot). The patient presented with an apparent OCA. Missense variants identified in *HPS3* (IP1) and *HPS5* (IP2) have to be classified as variant of uncertain significance (VUS), however we think it is valuable to report the findings of these variants in our patients.

### Mild Phenotype in BLOC-2 Deficiencies

For IP1 (HPS-3) two compound heterozygous missense variants in *HPS3* with predominantly and concordant disease-causing prediction were identified. Both variants are classified as VUS according to ACMG guidelines. The patient presented without cutaneous albinism, however, significantly hypopigmentation of the retina was detected in a specialized ophthalmological investigation. So far, the young girl exhibits mild bleeding symptoms. Taking these findings together these compound heterozygous variants most probably cause the patient’s mild phenotype. Such a mild phenotype has been described also in other patients with HPS-3 (Liu et al., 2021).

IP2 (HPS-5) carries a homozygous missense variant in *HPS5* (c.760G > T). The PP is concordant disease-causing. According to the ACMG criteria the variant classifies VUS. The patient does not exhibit apparent albinism, however, a life-long history of bleeding diathesis. None of her 8 children (all heterozygous carriers) are clinically affected as severely as their mother (IP2), although some show very minor bleeding symptoms, such as gingival bleeding and easy bruising. Because the patient lives outside of Germany, a specialized opthalmological investigation has not been performed to date. IP2 suffered from a cystic lesion of her gall bladder, therefore the gall bladder was removed. Clinically, she did not show any symptoms of colitis and the ultrasound investigations did not reveal any signs of colitis. Besides the *HPS5* variant, which we detected homozygous in the severely affected mother (IP2), we identified a heterozygous VUS in the gene *VPS33B*. According to OMIM alterations in the gene *VPS33B* are autosomal recessive associated with ARC (Arthrogryposis, renal dysfunction, and cholestasis). IP2 and the daughter who we investigated with NGS are carrier of the *VPS33B* variant; however, they do not show any symptoms of ARC. The son investigated with NGS presented wild type at the position. We did not investigate the other siblings for occurrence of the *VPS33B* variant. VPS33B is a protein essential for alpha granule biogenesis (Lo et al., 2005). Thrombin induced alpha-granule specific membrane protein P-selectin (CD62) exposure measured by flow cytometry was normal compared to healthy controls. However, a synergistic effect along with the homozygous *HPS5* variant, which is also classified as VUS is conceivable. We excluded Chediak-Higashi syndrome an autosomal recessive disease associated with alterations in the gene *LYST.* One of the features is a partial or severe reduction of dense-granules. *LYST* is included in our NGS gene panel and the analysis did not show any pathological findings. Inclusions in polymorph nuclear leukocytes have typically been reported in the blood smear of patients with Chediak-Higashi syndrome. IP2 did not show any inclusions in polymorph nuclear leukocytes and no signs of immunodeficiency.

Both *HPS3* and *HPS5* gene products are part of the BLOC-2 complex and patients primarily present with mild bleeding symptoms (Huizing et al., 2020; Nurden et al., 2020) and not necessarily with noticeable hypopigmentation (Liu et al., 2021). Complications usually did not include pulmonary fibrosis and immunodeficiency. Granulomatous colitis has been reported in about 10–20% of patients (Hussain et al., 2006). Most of the reported variants in *HPS3* and *HPS5* are variants with serious consequences like deletions, duplications, and variants affecting splicing (Huizing et al., 2020; Liu et al., 2021), only a few missense variants have been described (Huizing et al., 2004; Michaud et al., 2017; Lasseaux et al., 2018). It has also been described that two different nonsense variants in *HPS3* lead to different degrees of severities of OCA, suggesting a wide spectrum. One patient presented with a mild and two brothers exhibited a clear OCA (Sandrock-Lang et al., 2017; Lecchi et al., 2020). Both IP1 and IP2 show a mild phenotype, especially concerning the albinism. No further complications, like pulmonary fibrosis or immunodeficiency, were clinically observed in IP1 and IP2, so far.

### BLOC-1 Deficiency

The homozygous deletion of exon 6 in *DTNBP1*, found in IP3, has not been previously described. This deletion is pathogenic according to the ACMG guidelines due to the serious consequence. In addition, we showed the absence of dysbindin in platelet lysate. Although this patient has exhibited the characteristic HPS symptoms since birth, the genetic defect was detected rather late in life. Even if the symptoms of OCA are obvious, the diagnosis of HPS can be delayed if the bleeding diathesis is not recognized as part of the disease: the patient was 60 years old at the time when HPS-7 was diagnosed. To our knowledge only seven patients with HPS-7 have been reported worldwide comprising 4 nonsense or frameshift variants in *DTNBP1* (Li et al., 2003; Lowe et al., 2013; Bryan et al., 2017; Bastida et al., 2019). Due to the small number of patients, a definite statement regarding additional complications cannot be made. So far, only one patient has been reported developing granulomatous colitis (Lowe et al., 2013). The *DTNBP1* encoded Dysbindin-1 is involved in neurotransmission regulation and neurodevelopment (Tang et al., 2009; Ghiani et al., 2010) and seems to be involved in the etiology of Schizophrenia (Cheah et al., 2015; Wang et al., 2017). IP3 showed no symptoms of schizophrenia or other psychiatric disorders, however mild deficits in social interaction or depressive-like emotion are difficult to identify.

IP3 shows a history of severe and recurring airway infections which may possibly hint to immunodeficiency. The most obvious immune defect in lysosomal trafficking disorders is impaired NK cell and CTL cytotoxicity (Fischer et al., 2007). Strong degranulation defects predispose to hemophagocytic lymphohistiocytosis (HLH), a severe disorder of hyperinflammation, while the clinical consequences of milder defects are poorly characterized. A previous report has concluded that CTL cytotoxicity is normal in “sandy” mice (a model of HPS-7) (Bossi et al., 2005), which we could confirm in our patient. However, as previously documented in patients and mice with Chediak-Higashi-Syndrome (Jessen et al., 2011) the fresh NK cell degranulation assay is more sensitive than the CTL assay in detecting subtle impairments of the lytic machinery. Moreover, the degranulation defect could also affect other immune cells such as neutrophils or mast cells (Ramadass and Catz 2016), which could make an additional contribution to the observed infection susceptibility.

Two patients with HPS-9 (subunit of BLOC-1) have been reported presenting immunodeficiency (Badolato et al., 2012; Okamura et al., 2019). This might point to a possible complication in patients with BLOC-1 variants. However, only a few patients with BLOC-1 deficiencies have been described at all. More data are required to understand the role of BLOC-1 proteins in the immune system.

## Conclusion

Patients with variants of the BLOC-2 complex may not show the characteristic phenotype of a severe bleeding diathesis and oculocutaneous albinism; therefore, molecular genetic analysis including all HPS genes is needed for patients with a platelet delta granule secretion defect. Only a few patients with BLOC-1 deficiencies have been described so far and more data are needed to predict the phenotype and possible additional consequences. NGS can facilitate and accelerate the process of identifying the genetic defects regarding the different HPS-subtypes, especially if the patient presents with only a mild phenotype. Identification of a larger cohort for each subtype of HPS is important for the precise prediction of possible complications. This also offers new opportunities for further understanding the pathophysiology of HPS.

## Data Availability

The original contributions presented in the study are included in the article/Supplementary Materials, further inquiries can be directed to the corresponding author.
