# Supplementary material for: Hermansky-Pudlak Syndrome: Identification of Novel Variants in the Genes HPS3, HPS5, and DTNBP1 (HPS-7)
Source: Front Pharmacol. 2022 Jan 19;12:786937. doi: 10.3389/fphar.2021.786937 (PMC8807545; doi:10.3389/fphar.2021.786937)
Supplement: Supplementary file 1 [file DataSheet1.docx]

S1. Genes included in NGS Panel. Genes related to platelet disorders and albinism are in bolt. The probes designed for hybridization are covering all exons and exon/intron boundaries including the canonical splice sites (+/-25bp). For ANKRD26 we included the 5`UTR region to detect known regulatory pathogenic variants.

ACTN1, ADAMTS13, ANKRD26, ANO6, **AP3B1,** **AP3D1, BLOC1S3, BLOC1S5, BLOC1S6,** CD36, CD63, COL3A1, CYCS, DIAPH1, **DTNBP1**, ETV6, F8, FERMT3, FLI1, FLNA, GATA1, GFI1B, GNAS, GNE, GP1BA, GP1BB, GP5, GP6, GP9, HOXA11, **HPS1, HPS3, HPS4, HPS5, HPS6**, HRG, ITGA2, ITGA2B, ITGB3, JAK2, **LYST**, MASTL, MECOM, **MLPH**, MPL , MYB, MYH9, **MYO5A**, NBEA, NBEAL2, NFE2L2 (NRF2), ORAI1, P2RY12, PEAR1, PLA2G4A, PLAT, PLAU, PLG, PRF1, PRKACG, PROC, PROS1, **RAB27A**, RASGRP2, RBM8A, RUNX1, SEPTIN1, SEPTIN10, SEPTIN11 , SEPTIN12, SEPTIN14, SEPTIN2, SEPTIN3, SEPTIN4, SEPTIN5, SEPTIN6, SEPTIN7, SEPTIN8, SEPTIN9, SLFN14, SNAPIN, STAT3, STIM1, STX11, STXBP2, TBXA2R, TBXAS1, THPO, TUBB1, UNC13D, VIPAS39, VPS33B, VWF, WAS, WIPF1
